# Human retinal microvascular imaging using adaptive optics scanning light ophthalmoscopy

**DOI:** 10.1186/s40942-016-0037-8

**Published:** 2016-05-01

**Authors:** Toco Y. P. Chui, Shelley Mo, Brian Krawitz, Nikhil R. Menon, Nadim Choudhury, Alexander Gan, Moataz Razeen, Nishit Shah, Alexander Pinhas, Richard B. Rosen

**Affiliations:** 1 Department of Ophthalmology, New York Eye and Ear Infirmary of Mount Sinai, New York, NY USA; 2 Icahn School of Medicine at Mount Sinai, New York, NY USA; 3 Alexandria Faculty of Medicine, University of Alexandria, Alexandria, Egypt

**Keywords:** Retina, Adaptive optics, Blood vessels, Capillaries, Diabetic retinopathy, Sickle cell retinopathy, Retinal vein occlusion, Fluorescein angiography

## Abstract

**Background:**

Retinal microvascular imaging is an especially promising application of high resolution imaging since there are increasing options for therapeutic intervention and need for better structural and functional biomarkers to characterize ocular and systemic vascular diseases.

**Main body:**

Adaptive optics scanning light ophthalmoscopy (AOSLO) is an emerging technology for improving in vivo imaging of the human retinal microvasculature, allowing unprecedented visualization of retinal microvascular structure, measurements of blood flow velocity, and microvascular network mapping. This high resolution imaging technique shows significant potential for studying physiological and pathological conditions of the retinal microvasculature noninvasively.

**Conclusion:**

This review will briefly summarize the abilities of in vivo human retinal microvasculature imaging in healthy controls, as well as patients with diabetic retinopathy, retinal vein occlusion, and sickle cell retinopathy using AOSLO and discuss its potential contribution to scientific research and clinical applications.

## Background

The retina has one of the highest metabolic demands per unit weight of any tissue in the human body [1], making it especially vulnerable to disease processes that damage the vascular network and lessen the oxygen and nutrient supply to the tissue. The ability to detect these disease manifestations early is important as it offers the opportunity to intervene systemically as well as locally in order to slow, stop or even reverse these changes. Macroscopic features of the retinal vasculature, such as arteriolar narrowing, arteriovenous nicking, hemorrhages and microaneurysms have traditionally been used as signs of progressive cardiovascular disease, as well as hypertension and diabetes mellitus. Studying microscopic features of the retinal vasculature would theoretically enable earlier detection of disease. Thus, our ability to image retinal microvasculature is important for providing better knowledge of the normal physiological retina and pathological process, allowing development of new strategies to prevent or delay disease progression.

Recently, advances in high resolution imaging techniques such as adaptive optics and optical coherence tomography angiography (OCTA) have expanded our ability to map the living human retinal vasculature noninvasively without the use of exogenous contrast agents [2–8]. In particular, adaptive optics scanning light ophthalmoscopy (AOSLO) is an emerging technology for the visualization of microscopic structures in the living human retina to an extent that had not been previously possible with conventional clinical imaging modalities [9]. This imaging technique utilizes deformable mirrors to correct for ocular aberrations, allowing high resolution non-invasive imaging of retinal structures including retinal nerve fibers, retinal microvasculature, photoreceptors, retinal pigment epithelium, and lamina cribrosa [4, 9–18]. Specifically, the non-invasive nature of AOSLO imaging makes it very appealing for screening, detecting, and monitoring subclinical microvascular changes in the human retina, which may enable earlier intervention against retinal diseases.

## Review

### AOSLO imaging with confocal and nonconfocal detection schemes

#### Confocal detection schemes

**Fig. 1:**
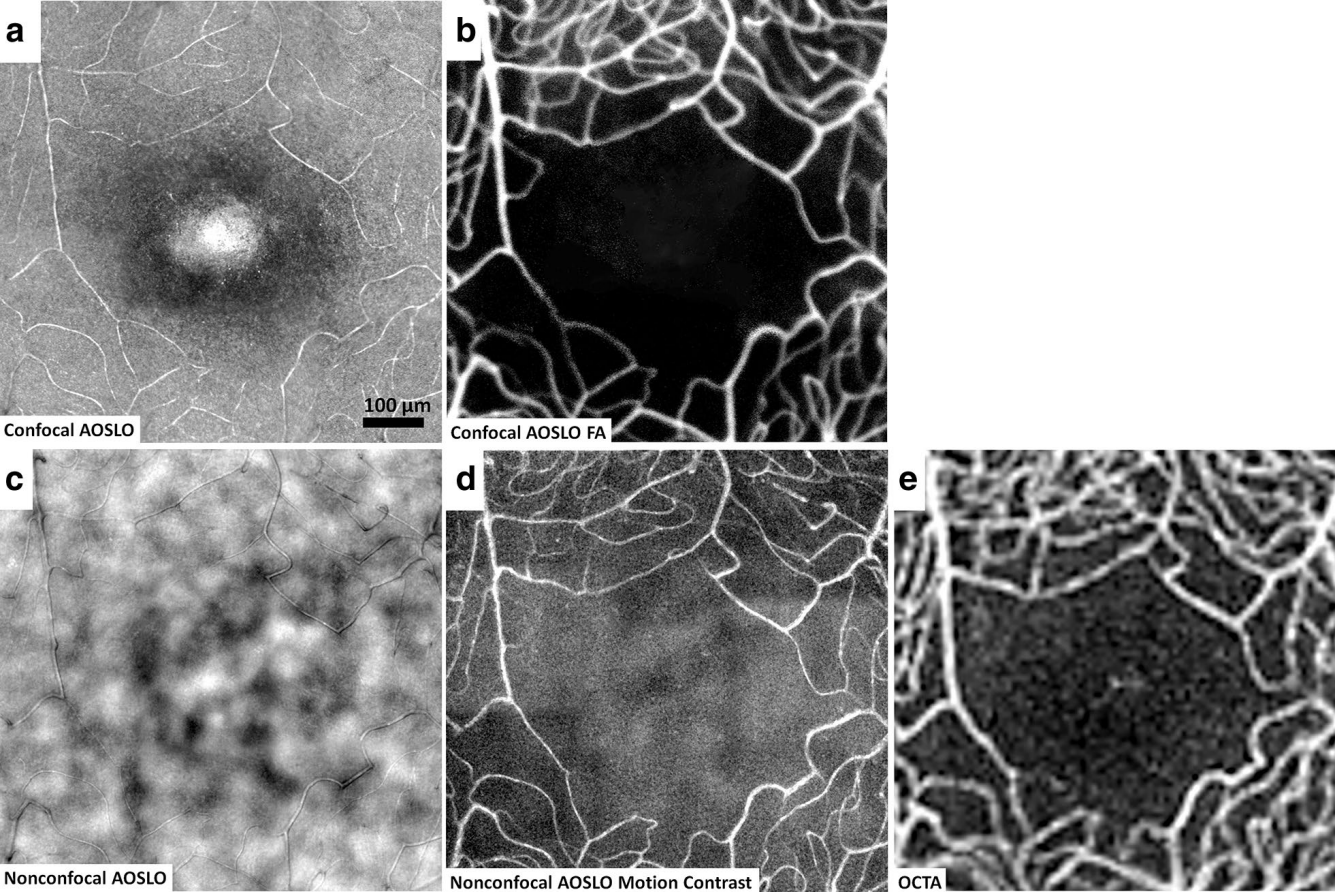
Visualization of foveal capillary network and foveal avascular zone in a 25-year-old healthy male control (RR0188) using different imaging modalities. **a** Confocal AOSLO structural image, **b** confocal AOSLO FA perfusion map, **c** nonconfocal AOSLO structural image, **d** motion contrast perfusion map generated using registered nonconfocal AOSLO structural movies, and **e** OCTA imaged using Optovue RTVue XR Avanti (AngioVue, Optovue, Inc., Fremont, CA)

Traditionally, confocal AOSLO has allowed direct visualization of microscopic structures of the human retina with unprecedented contrast and resolution [9, 10, 13, 14, 19]. This confocal imaging technique is achieved by placing a spatial pinhole at a retinal conjugate plane to include only direct backscattered light and eliminate multiply scattered light (out-of-focus) simultaneously. Non-invasive assessment of the retinal microvascular network using confocal AOSLO has been demonstrated on healthy and diseased retinas [3, 18, 20]. This technique has been applied successfully at the foveal region (Fig. 1a) where there is simpler anatomy and few capillary layers. It has limited success, however, more peripherally where the capillary meshwork is more complex, such as in the peripapillary and perifoveal regions. In addition, the strong direct backscattering signal from highly reflective tissues, such as the retinal nerve fiber layer and glial cells, masks the backscattered light from microvasculature, making it more difficult to image using this technique in the periphery [16]. Fluorescein angiography (FA), coupled with confocal AOSLO, has enhanced the success of imaging the retinal microvasculature of the macula (Fig. 1b) and peripapillary retina [21, 22]. Although confocal AOSLO FA is considered safe and has been used to show perfusion and non-perfusion of the retinal capillaries in healthy controls and patients with vasculopathies [23–25], it requires an additional light source for fluorescein excitation and administration of oral fluorescein, making it more invasive with some risk of patient discomfort and potential side effects such as nausea and itching.

#### Nonconfocal detection schemes

**Fig. 2:**
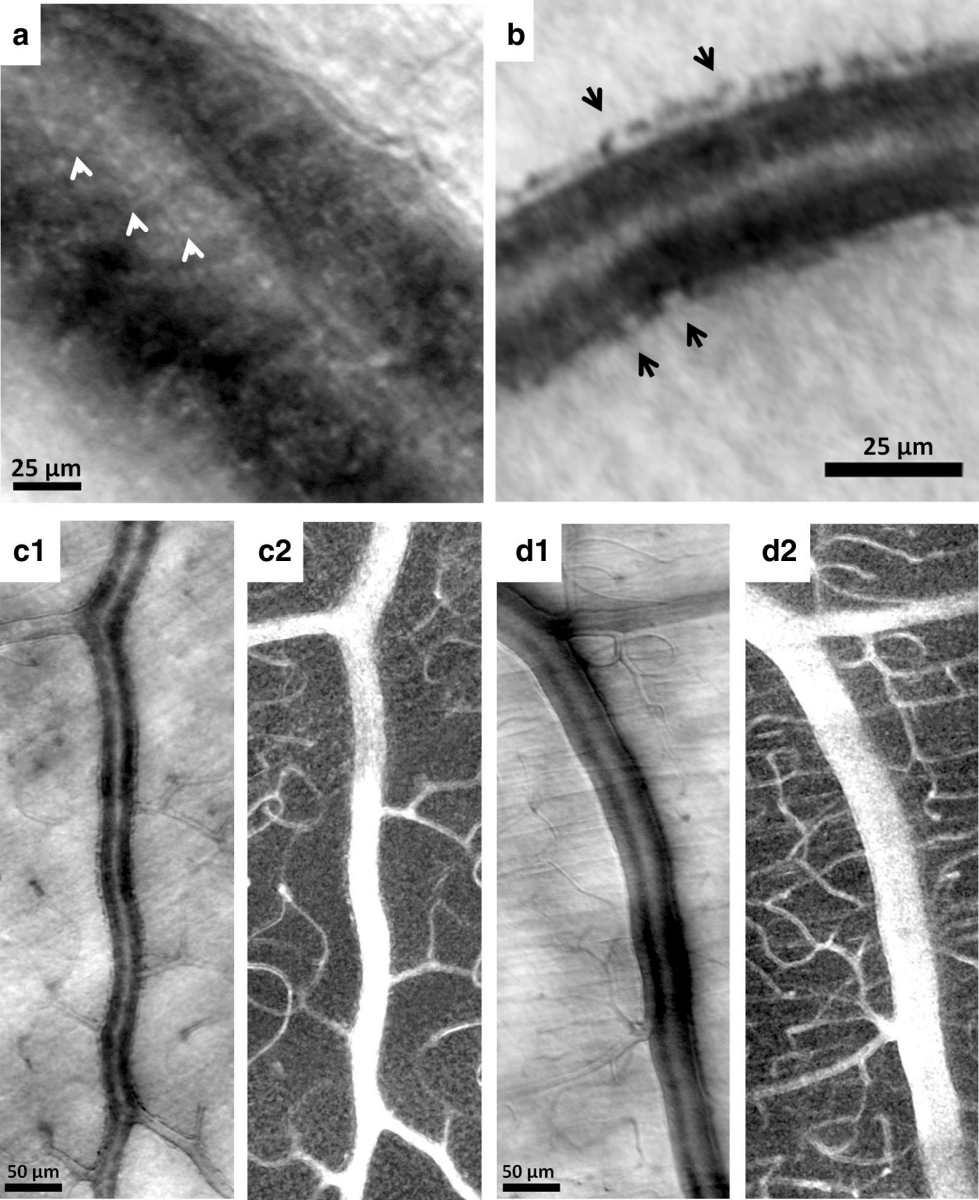
*In vivo* imaging of human retinal vascular wall fine structures in arterioles and venules using nonconfocal AOSLO. Structural images of **a** venule and **b** an arteriole of a 25-year-old male healthy control (RR0216). *Arrows* indicate individual vascular mural cells. **c1** Structural image of a 40 µm arteriole located at 5° superior to the fovea in a 26-year-old female (RR0172). **c2** Corresponding motion contrast perfusion map of **c1**. Periarteriolar capillary free zone along the arteriole is clearly visualized. **d1**, **d2** Structural image and motion contrast perfusion map of a 50 µm venule located at 7° superior to the fovea in a 26-year-old male (RR0025). No distinct capillary free zone along the venule is observed

**Fig. 3:**
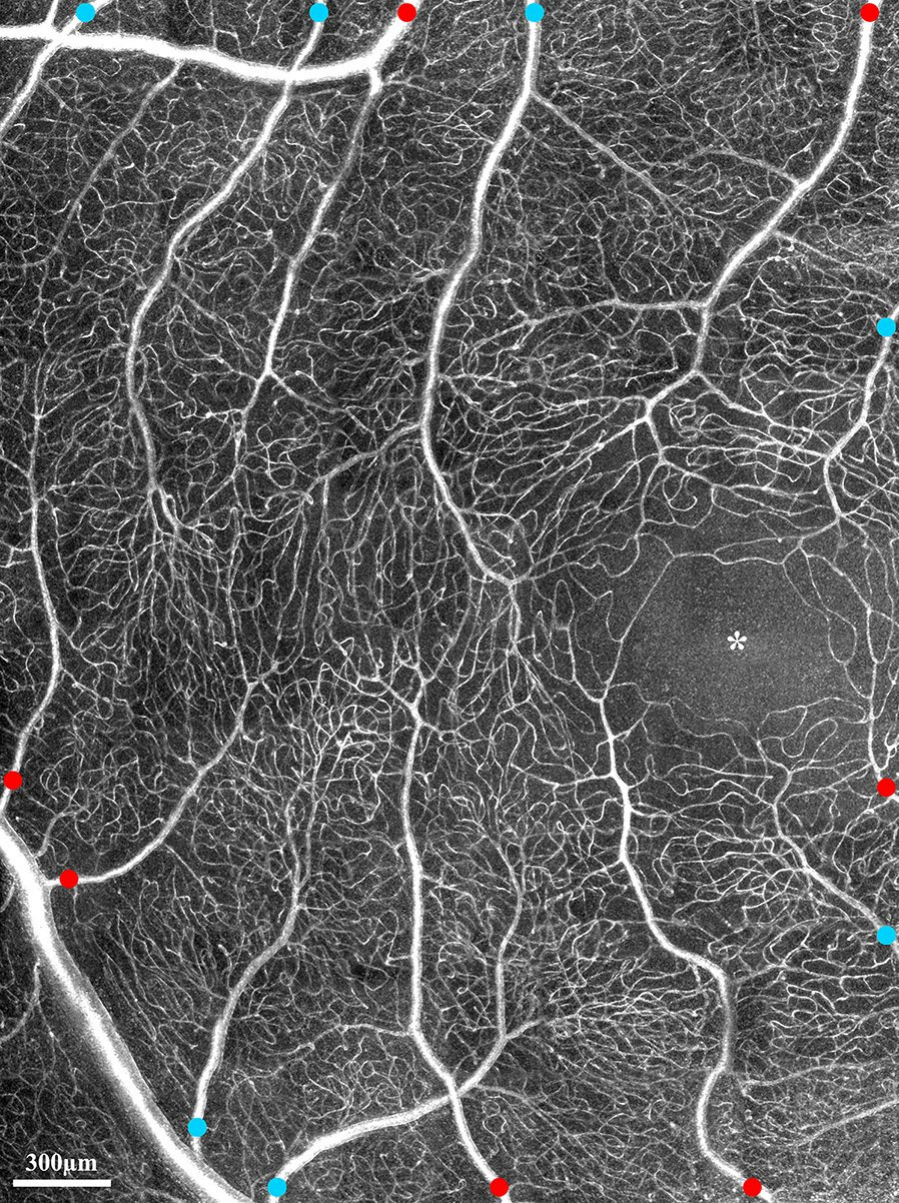
Motion contrast perfusion map of the right eye of a 35-year-old healthy male control generated using nonconfocal AOSLO registered movies. Connections between arterioles and venules are readily visible. *Asterisk* indicates the foveal avascular zone. Arterioles and venules are marked in *red* and *blue*, respectively. Image courtesy of Stephen A Burns and Dean A Vannasdale, Indiana University [30]

Recently, the options of adaptive optics microscopic imaging have been expanded through the use of multiply scattered light with nonconfocal detection schemes (e.g. offset pinhole, dark field, and split detection). The general principles and applications of this technique have been described in detail elsewhere [15, 16, 26–30]. Several studies have demonstrated that multiply scattered light, which is systematically removed in confocal AOSLO imaging, contains valuable information about the retina. Detection of multiply scattered light allows non-invasive visualization of retinal vascular structures using reflectance imaging (Fig. 1c) and provides microvascular perfusion maps through the use of motion contrast image processing (Fig. 1d) [2, 12, 16, 29, 31]. Microvascular perfusion maps are generated by extraction of moving elements from registered nonconfocal AOSLO videos, such as the intravascular flow of erythrocytes against the background of static tissue [2]. Nonconfocal AOSLO imaging provides structural images and perfusion maps comparable to those of confocal AOSLO FA with additional structural information such as fine structural components of vascular walls [22]. Figure 2 shows in vivo visualization of retinal vascular mural cells (Fig. 2a, b) and motion contrast perfusion maps (Fig. 2c, d) in arterioles and venules using nonconfocal AOSLO. Connections between arterioles and venules at the fovea and the temporal retina are readily seen on widefield motion contrast perfusion maps created by montaging as illustrated in Fig. 3.

### Comparison of perfusion maps—IVFA, AOSLO, and OCTA

Since it was introduced by Novotny and Alvis in 1961, intravenous fluorescein angiography (IVFA) has been the clinical gold standard for assessing retinal vascular disease [32]. It produces an analyzable picture of the retinal vasculature in vivo and is able to detect pathological features such as non-perfusion, infarctions, edema, neovascularization, microaneurysms and leakage at blood–retinal barrier ruptures [21, 33]. It has helped with diagnosis, as well directing treatment of a variety of conditions ranging from diabetic macular edema to retinopathy of prematurity [34, 35]. While IVFA has fostered many discoveries about retinal vasculopathies, it has some limitations. It offers a widefield view of the vasculature, but has inherent limitations in axial and lateral resolution. IVFA has difficulty revealing capillaries with smaller diameters, and those that are anatomically deeper and further from the fovea, as suggested by examination of comparable flat-mounted histological sections [36]. In addition, there is concern about the invasive nature of IVFA due to the use of an exogenous contrast agent, which occasionally produces minor reactions such as nausea and pruritus, and much more rarely anaphylaxis and death [37–41].

**Fig. 4:**
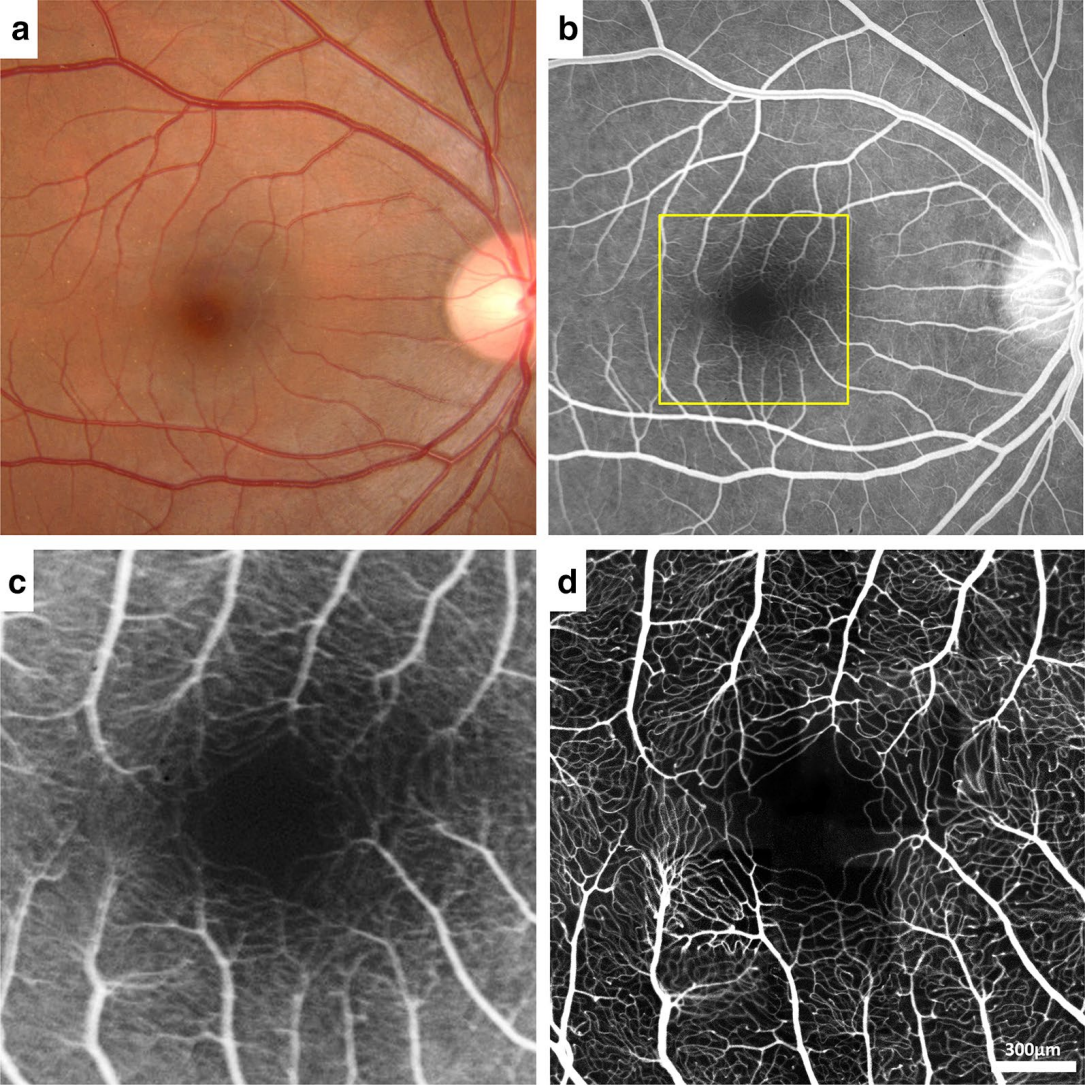
Comparison of IVFA and confocal AOSLO FA perfusion map in a healthy control. Images of the right eye of a 35-year-old healthy male control (RR0001). **a** Conventional fundus photograph. **b** IVFA. *Yellow box* indicates the region imaged with confocal AOSLO FA. **c** Magnified IVFA compared to **d** the same region imaged with confocal AOSLO FA. **c**, **d** Reproduced with permission from Pinhas et al. [21]

**Fig. 5:**
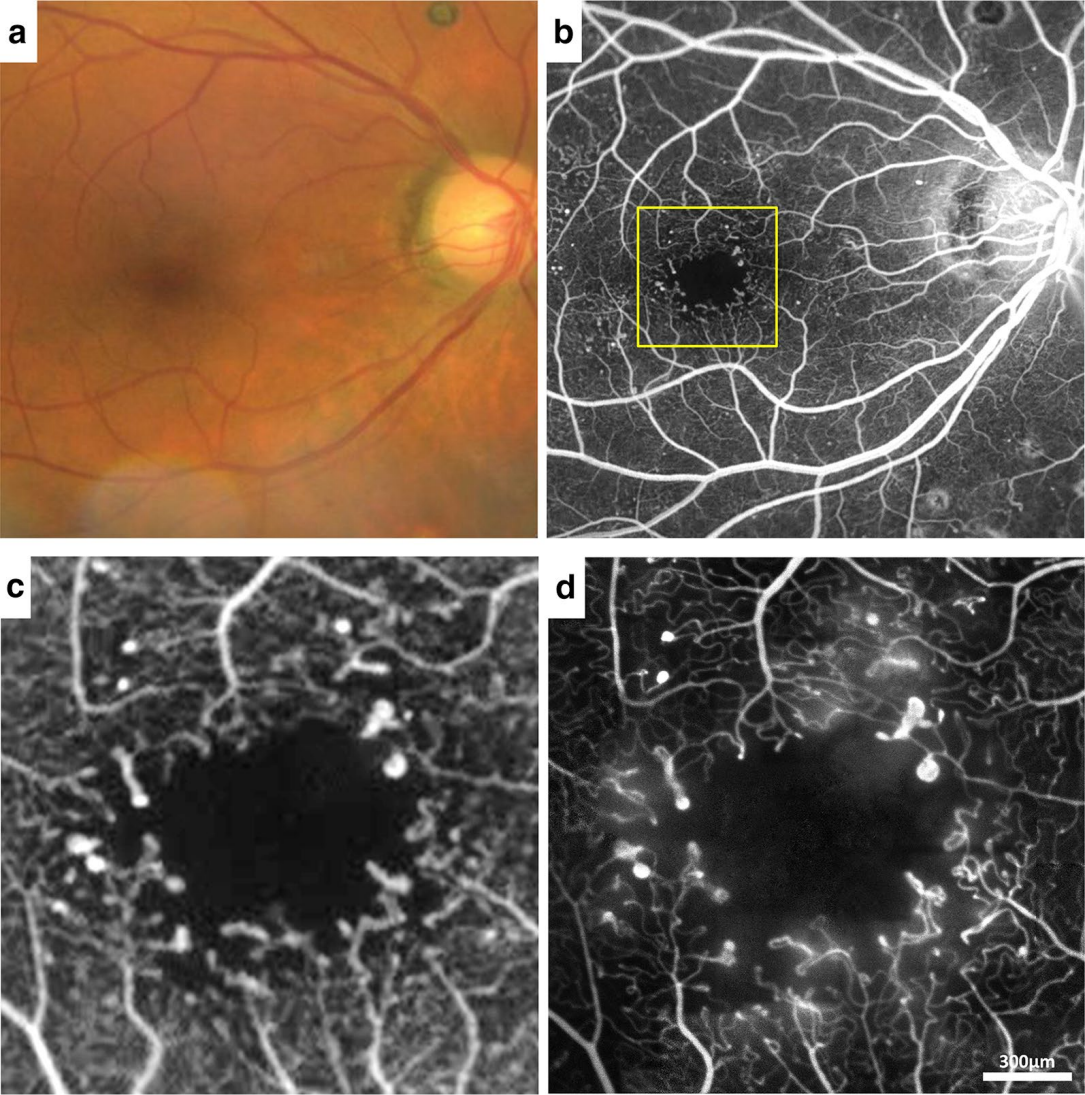
Comparison of IVFA and confocal AOSLO FA perfusion map in proliferative diabetic retinopathy. Images of the right eye of a 50-year-old male with proliferative diabetic retinopathy (RR0265). **a** Conventional fundus photograph. **b** IVFA shows numerous microaneurysms scattered around the macular region. *Yellow box* indicates the region imaged with confocal AOSLO FA. **c** Magnified IVFA compared to **d** the same region imaged with confocal AOSLO FA

Confocal AOSLO FA performed with oral administration of fluorescein has an ability to image the microscopic detail of the retinal vasculature with greater resolution and contrast than with conventional imaging [21]. Comparisons of IVFA and confocal AOSLO FA perfusion map in a healthy control and a patient with proliferative diabetic retinopathy are included in Figs. 4 and 5, respectively. Confocal AOSLO FA offers great potential for precise quantification of the foveal avascular zone, vessel lumen diameters, branching patterns, capillary density and tortuosity in both healthy and diseased retinas. Oral administration of fluorescein with confocal AOSLO FA enables extended fluorescein imaging time with lower risk of side effects, but the small imaging field of view of confocal AOSLO FA necessitates montaging to create larger perfusion maps.

Nonconfocal AOSLO coupled with motion contrast processing reveals maps of retinal microvasculature perfusion with detail comparable to confocal AOSLO FA, but without the need for any exogenous contrast agent [22]. This image processing technique takes advantage of the motion of multiply scattering particles, in this case, intravascular erythrocytes, which serve as intrinsic markers revealing the perfusion status of retinal microvasculature [2, 29]. Limitations of this technique include motion artifacts, inability to visualize fluorescein leakage or pooling, and difficulty in detecting blood vessels with slow or intermittent perfusion in comparison to IVFA and confocal AOSLO FA.

OCTA imaging is a new and emerging technology based on motion contrast with widespread clinical potential for mapping the retinal vasculature, detecting retinal vascular abnormalities and monitoring disease progression (Fig. 1e). Similar to nonconfocal AOSLO, OCTA is completely non-invasive, not requiring an exogenous contrast agent. In comparison to adaptive optics imaging techniques, OCTA’s major advantage is the much shorter imaging time. OCTA also has a major advantage over IVFA or confocal AOSLO FA, since it is able to delineate the different layers of retinal capillary beds including the choriocapillaris in a single scan [22]. However, since it relies on motion contrast, it is subject to projection artifacts from more superficial vessels shadowing upon the deeper layer vessels, more prone to motion artifacts, and is unable to show leakage or slowed perfusion. Both nonconfocal AOSLO and OCTA provide attractive alternatives to IVFA or confocal AOSLO FA, since they allow frequent non-invasive evaluation and follow up exams.

Despite their advantages, AOSLO and OCTA are relatively new to the clinic and not yet considered routine techniques for imaging retinal vasculature. As with any new technology, the accuracy and reproducibility of AOSLO and OCTA must be tested in order to establish their validity and suitability for routine clinical implementation. These investigations are especially critical prior to initiation of cross-sectional or longitudinal studies of pathological microvascular change. Since accuracy and reproducibility have yet to be established, such studies must be conducted to define normative anatomic and physiologic standards before we can reliably assess disease states. In addition, comparative analyses between AOSLO and OCTA may be instructive regarding the significance of vascular patterns observed and their relationship to various vascular abnormalities.

### Clinical applications of retinal microvascular imaging using AOSLO

**Fig. 6:**
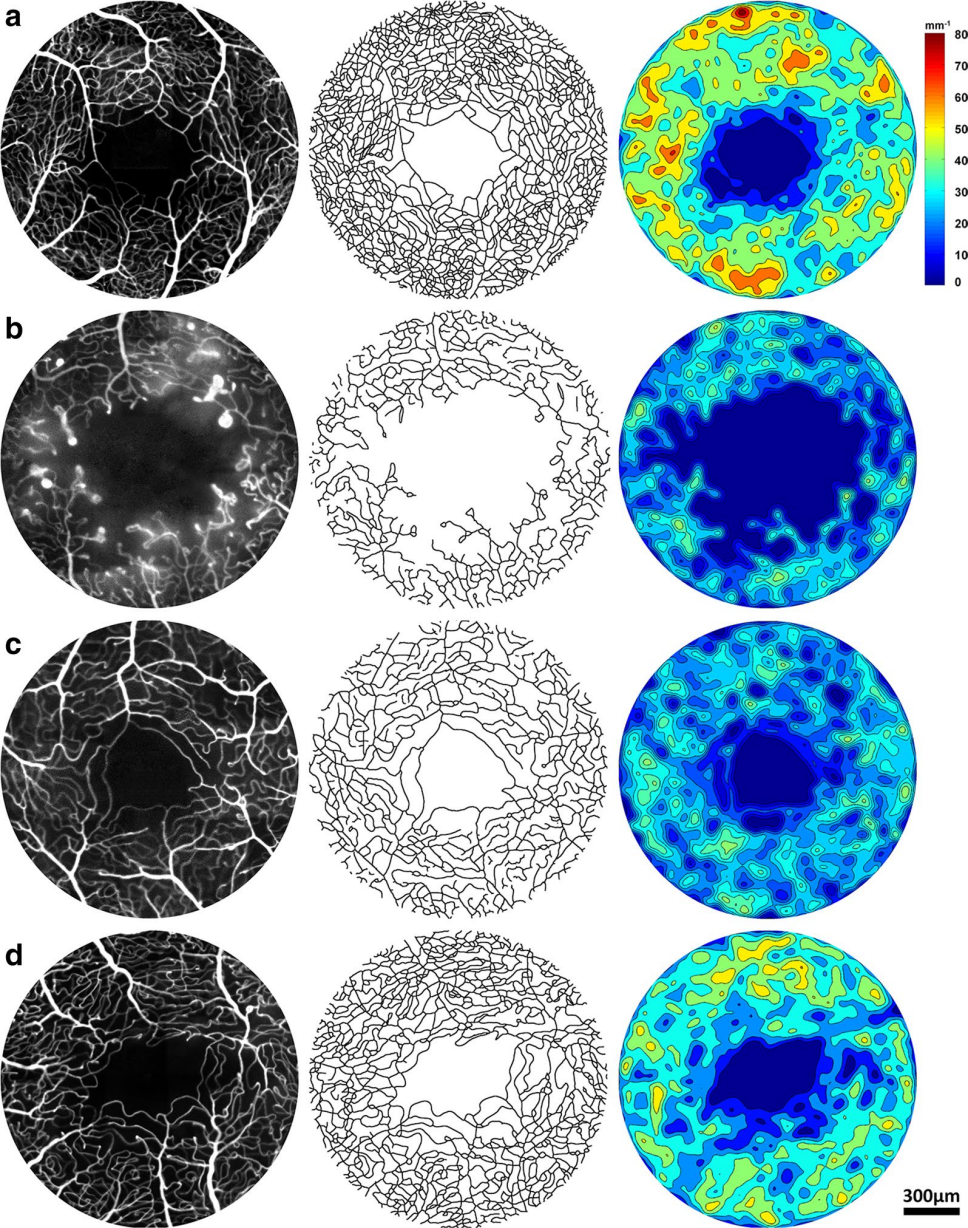
Confocal AOSLO FA perfusion maps (*left column*), skeletonized perfusion maps (*middle column*), and colorized vessel density contour maps (*right column*) in healthy and diseased retinas. **a** The left eye of a 25-year-old healthy male control—RR0188, **b** the right eye of a 50-year-old male with proliferative diabetic retinopathy—RR0265, **c** fellow eye (right eye) of a 46-year-old male with branch retinal vein occlusion—RR0272, and **d** the left eye of a 44-year-old female with sickle cell retinopathy—RR0204

Currently, there are a variety of cross-sectional AOSLO studies which describe the structural and functional changes to the retinal capillaries in patients with vasculopathies [20, 22–25, 42–47]. This section briefly discusses the AOSLO imaging characteristics of three common retinal vasculopathies: diabetic retinopathy, retinal vein occlusion, and sickle cell retinopathy. Information obtainable using confocal and nonconfocal AOSLO includes foveal avascular zone geometry, vessel density, vascular lumen diameter, vessel wall thickness, vascular mural cells, capillary perfusion status, capillary tortuosity, and microaneurysm morphology; all of which can be used to describe the variety of retinal physiologic and pathophysiologic processes. Examples of confocal AOSLO FA perfusion maps of healthy and vasculopathic eyes, and their corresponding colorized vessel density contour maps are displayed in Fig. 6.

#### Diabetic retinopathy

**Fig. 7:**
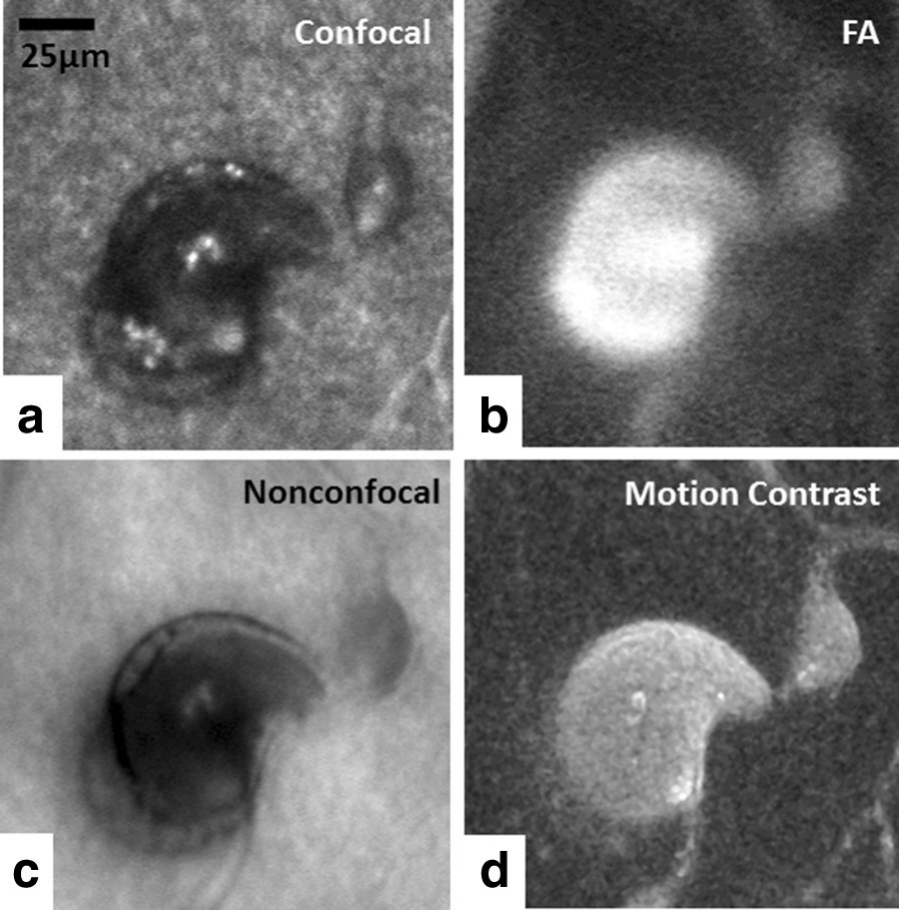
A retinal microaneurysm in a 62-year-old female with non-proliferative diabetic retinopathy imaged with different AOSLO modalities (RR0217). **a** Confocal AOSLO structural image, **b** confocal AOSLO FA perfusion map, **c** nonconfocal AOSLO structural image, and **d** nonconfocal AOSLO motion contrast perfusion map

Diabetic retinopathy is the most prevalent retinal vascular disease, causing visual impairments in 7.6 million Americans age 40 and older [48]. It typically develops over several years and may remain asymptomatic until vision-related complications occur. Clinical diagnosis of diabetic retinopathy has traditionally relied on the detection of microangiopathy such as microaneurysms, exudates, and hemorrhages using ophthalmoscopy, conventional fundus photography, and IVFA. Histologic studies have shown that the onset of diabetic retinopathy is characterized by pericyte loss and basement membrane thickening consequent to prolonged hyperglycemia [49–51]. These early changes however are not visible with the current generation clinical imaging modalities, and are thus considered subclinical. A number of studies using AOSLO imaging have already demonstrated the ability to detect these subclinical changes in vivo [20, 42, 43, 52]. Quantification of vascular changes is critical, because there is growing evidence that the retina changes in structure and function in aging healthy individuals and patients with early diabetic retinopathy [20, 30, 53, 54]. Using confocal AOSLO, Tam et al. showed greater arteriovenous tortuosity surrounding the fovea in a group of 15 type 2 diabetic patients without retinopathy, when compared to healthy controls [20]. Burns et al. evaluated perifoveal capillary diameters in 7 patients with mild to moderate non-proliferative diabetic retinopathy using nonconfocal AOSLO [42]. Their results demonstrated that with subclinical microvascular remodeling, capillary diameter and arteriolar wall thickness were significantly larger in the diabetic group when compared to the controls. These measureable structural changes represent potential clinical biomarkers for screening diabetics for early retinopathy, allowing the opportunity to detect changes earlier and guide better management of disease. Recently our group demonstrated that successful identification and quantification of retinal microvascular abnormalities such as microaneurysms and capillary dropout can be obtained using confocal AOSLO FA perfusion maps (Fig. 6b) [23, 25]. Dubow et al. showed that the combination of confocal AOSLO structural images and confocal AOSLO FA perfusion maps could be useful for evaluating retinal microaneurysm morphology and geometry and for enhancing our understanding of the disease process [25]. Figure 7 shows a saccular microaneurysm imaged using different AOSLO modalities in a 62-year-old patient with non-proliferative diabetic retinopathy. Microaneurysm wall structure is readily visible on the confocal (Fig. 7a), and nonconfocal (Fig. 7c) AOSLO structural images, permitting a rich survey of structural and functional features of microaneurysms seen with different techniques. While the confocal AOSLO FA perfusion map shows fluorescein pooling in the stagnant end of the lumen (Fig. 7b), the nonconfocal AOSLO motion contrast perfusion map isolates the region of active blood flow (Fig. 7d).

#### Retinal vein occlusion

Retinal vein occlusion is second only to diabetic retinopathy as a cause of retinal vascular morbidity [55]. Branch retinal vein occlusion has a prevalence of 5.20 per 1000, being more common than central retinal vein occlusion [56]. Clinical presentations of retinal vein occlusion may range from asymptomatic to moderate or even severe loss of vision. Chronic vascular changes include abnormal or absence of perfusion distal to the point of occlusion, vessel leakage, dilated collateral vessels, and neovascularization [55, 57].

**Fig. 8:**
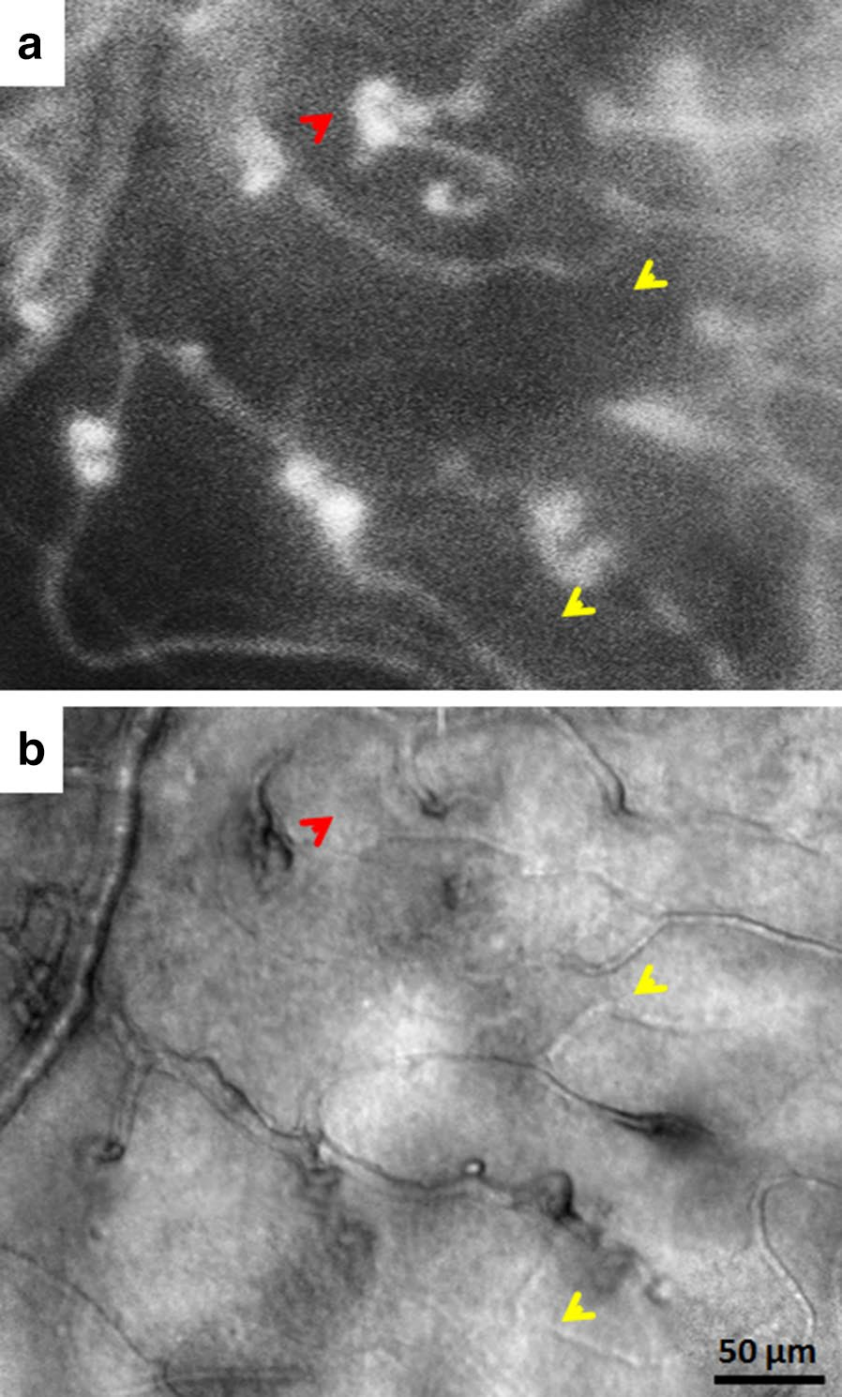
Imaging of non-perfused capillaries and microaneurysms in a 24-year-old male with hemiretinal vein occlusion using AOSLO (RR0235). **a** Confocal AOSLO FA perfusion map obtained at 3° superior to the fovea in the right eye (affected eye). **b** Visualization of the non-perfusion capillaries on nonconfocal AOSLO structural image. Comparison of the confocal AOSLO FA perfusion map and nonconfocal AOSLO structural image reveals non-perfused capillaries (*yellow arrows*) and a microaneurysm located at the deep layer (*red arrows*)

Currently, there are only few AOSLO studies on retinal vascular occlusion. Using a commercially-available confocal AOSLO, Akagi-Kurashige et al. observed that retinal capillaries were more dilated and tortuous in patients with branch retinal vein occlusion [58]. In patients with nonischemic central retinal vein occlusion, Pinhas et al. reported on the foveal vessel density in 4 affected and 10 fellow eyes using confocal AOSLO FA [24]. While vessel density of affected eyes was expectedly lower than that of fellow or control eyes, fellow eyes demonstrated vessel densities which were significantly reduced compared to healthy controls (Fig. 6c). This subclinical vascular change observed in asymptomatic fellow eyes may have important implications on the pathophysiology of venous occlusions and may explain the increased lifetime risk of occlusion in fellow eyes. Additionally, our recent efforts to compare confocal AOSLO FA perfusion maps and nonconfocal AOSLO structural images in patients with retinal vein occlusion have revealed an array of microangiopathic features such as non-perfused capillaries, leakage, and microaneurysms (Fig. 8), not fully appreciated from conventional imaging, which may provide additional insight into the full microanatomic impact of this disease.

#### Sickle cell retinopathy

**Fig. 9:**
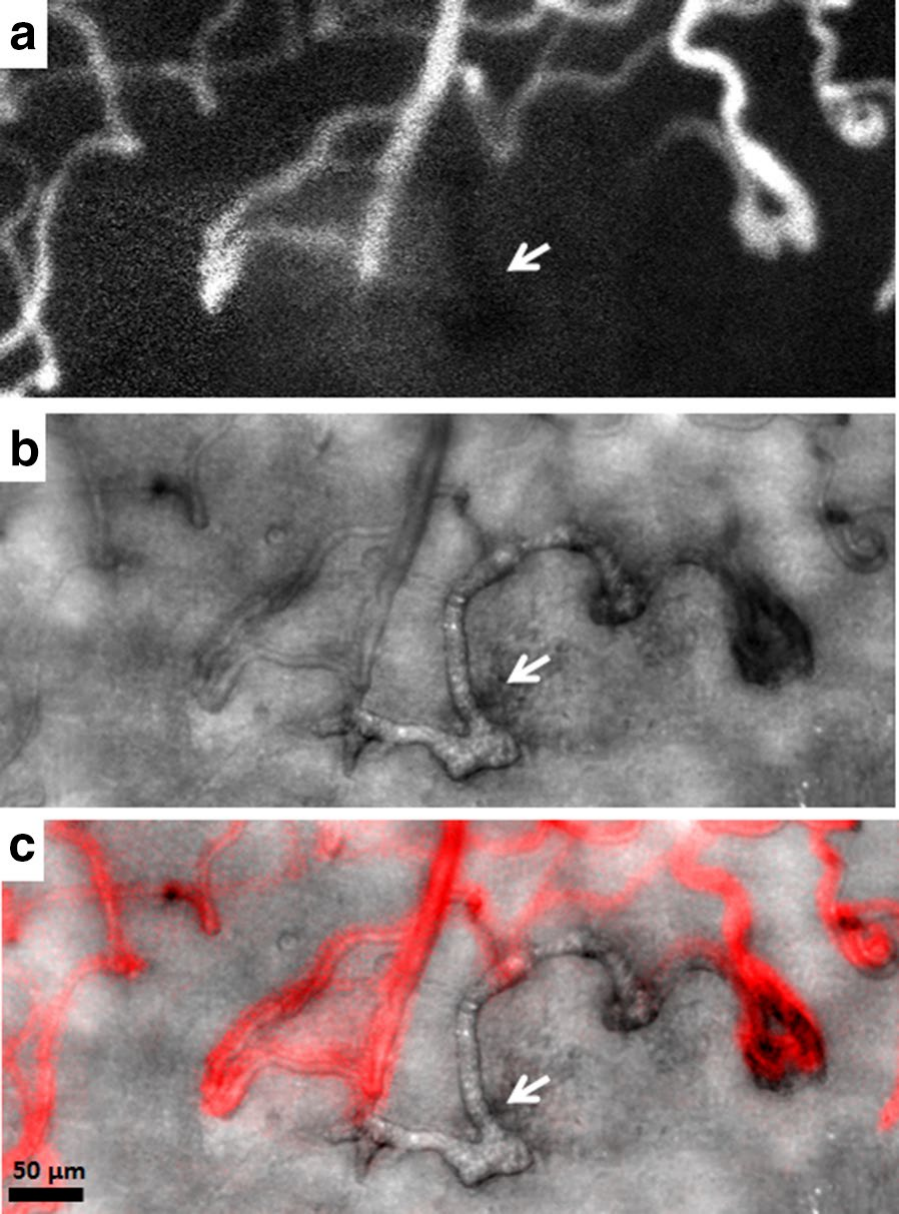
Imaging of non-perfused blood vessels in a 24-year-old male with sickle cell retinopathy using AOSLO (RR0292). **a** Confocal AOSLO FA perfusion map obtained at 2° superior to the fovea in the right eye. Shadow of a non-perfused blood vessel is indicated by a *white arrow*. **b** Visualization of the non-perfusion blood vessel on nonconfocal AOSLO structural image. **c** Superimposed composite of **a** (in *red*) and **b** reveals the location of perfused and non-perfused blood vessels

Sickle cell retinopathy is a major complication of sickle cell disease that can lead to visual impairment. Sickle cell disease is caused by a point mutation on the beta globin gene, which limits erythrocyte deformity and reduces membrane elasticity [59]. These rigid erythrocytes have a tendency to stack and occlude small blood vessels of nearly any organ, including the retinal capillaries. Clinical presentations of non-proliferative sickle cell retinopathy include hemorrhages, venous tortuosity, and enlargement of the foveal avascular zone [60]. On the other hand, proliferative sickle cell retinopathy reflects more advanced disease and is characterized by retinal neovascularization in response to ischemia following occlusion, especially in the peripheral retina. These new blood vessels are vulnerable to rupture from vitreous traction, leading to vitreous hemorrhage and increased risk of retinal detachment [61]. Recent application of confocal AOSLO FA to imaging patients with sickle cell retinopathy has demonstrated significantly higher capillary tortuosity compared to healthy controls [47]. Further studies exploring the clinical application of capillary tortuosity as a metric for monitoring sickle cell retinopathy progression are currently underway. Examples of increased capillary tortuosity and non-perfusion are shown in Fig. 6d and Fig. 9, respectively. Although the identification of non-perfused capillaries is clearly demonstrated by comparing the confocal AOSLO FA perfusion map and the nonconfocal AOSLO structural image (Fig. 9, white arrows), only the nonconfocal AOSLO image reveals the structural appearance of the non-perfused capillaries, which are clearly absent from the confocal AOSLO FA perfusion map.

### Longitudinal imaging of retinal microvasculature using AOSLO

**Fig. 10:**
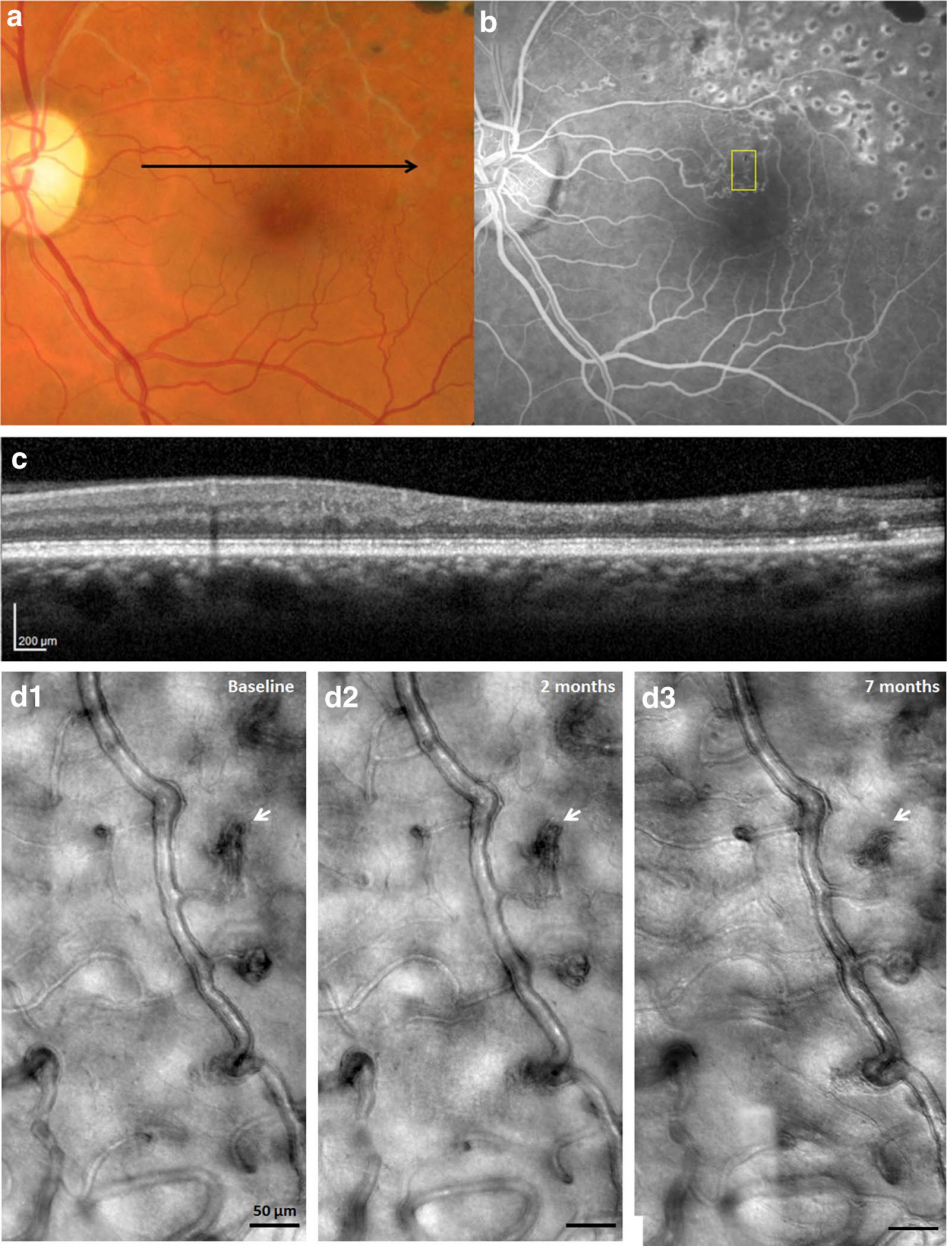
Clinical photographs and nonconfocal AOSLO images of a 55-year-old female with branch retinal vein occlusion (RR0129). **a** Fundus photograph of the left eye. *Black arrow* indicates location of horizontal SDOCT scanning. **b** IVFA showing tortuous blood vessels with superior temporal region treated with pan-retinal photocoagulation. *Yellow box* indicates the region imaged with nonconfocal AOSLO. **c** Horizontal SDOCT ~2.5° superior to the fovea obtained at the first AOSLO imaging visit. **d** Longitudinal nonconfocal AOSLO imaging performed at baseline, 2 months, and 7 months shows only minor microvascular changes across visits (*white arrows*). Panel D1 was reproduced with permission from Chui et al. [22]

**Fig. 11:**
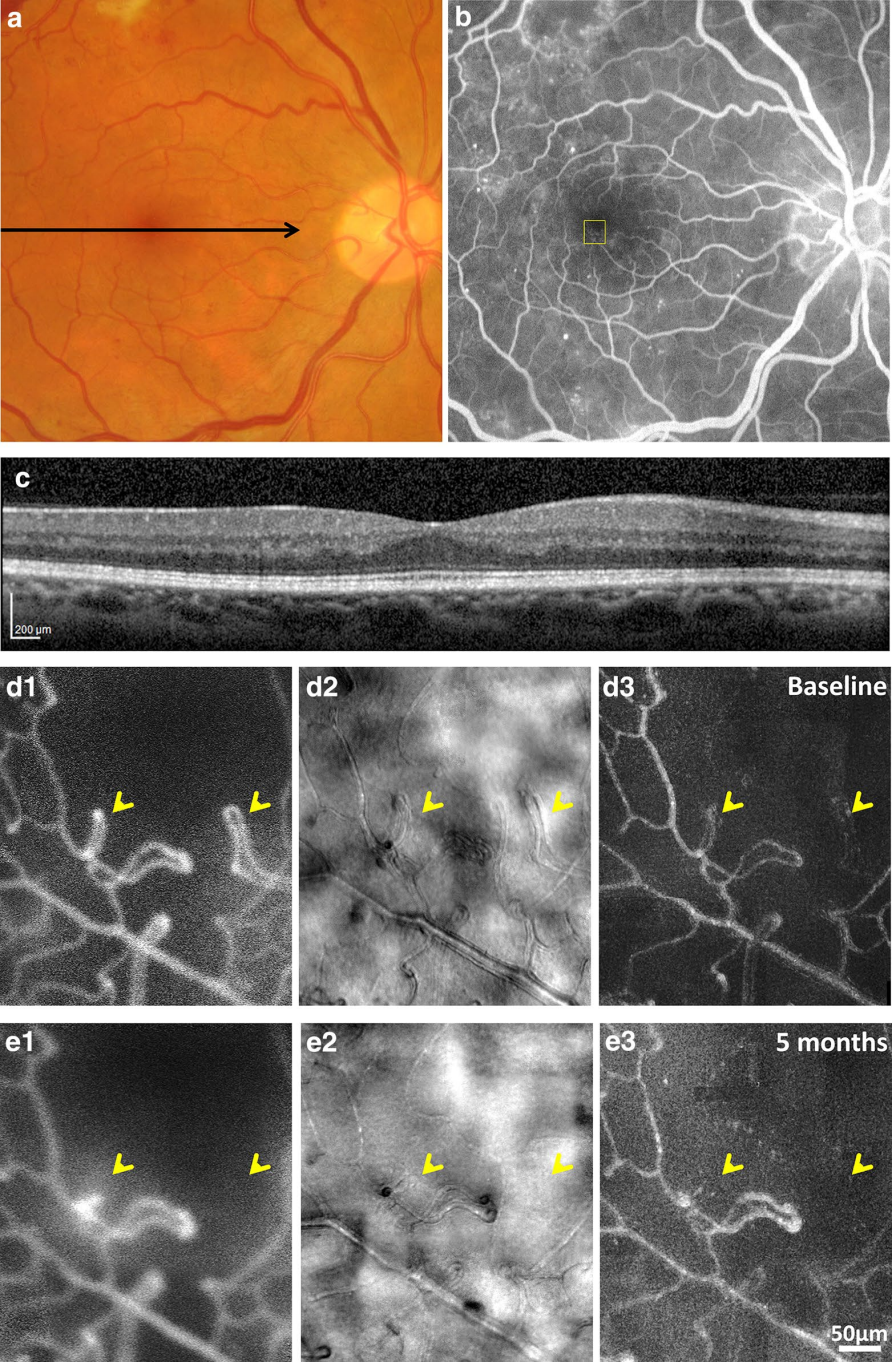
Clinical photographs and AOSLO images of a 49-year-old female with proliferative diabetic retinopathy (RR0167). **a** Fundus photograph of the right eye. *Black arrow* indicates location of horizontal foveal SDOCT scanning. **b** IVFA showing scattered microaneursyms and fluorescein leakage. *Yellow box* indicates the region imaged with AOSLO. **c** Horizontal foveal SDOCT obtained at the first AOSLO imaging visit. Longitudinal AOSLO imaging performed at **d** baseline and **e** 5 months. **d**1, **e**1 Confocal AOSLO FA perfusion maps. **d**2, **e**2 Nonconfocal AOSLO structural images. **d**3, **e**3 Nonconfocal AOSLO motion contrast perfusion maps. *Yellow arrows* indicate capillary non-perfusion and regression over 5 months

As revealing as AOSLO cross-sectional imaging has been for displaying the details of microvascular change, its capability for non-invasive recording of longitudinal data is even more extraordinary, providing insight into the natural progression of microangiopathies. The ability to track capillary perfusion and microaneurysms longitudinally allows the opportunity to develop metrics of vascular change and apply them as biomarkers to help stage disease progression and assess treatment response [62–66]. Figures 10 and 11 present two examples of longitudinal imaging of retinal microvasculature using AOSLO, which demonstrate its utility in documenting dynamic structural and functional changes [43, 52].

Figure 10 shows fundus photographs and nonconfocal AOSLO structural images of a 55-year-old female with branch retinal vein occlusion of 4 years duration. She received segmental scatter photocoagulation in the left eye 4 years prior to her baseline AOSLO imaging session. Nonconfocal AOSLO structural images of the left eye were obtained at baseline, 2 months, and 7 months. During this period, her visual acuity in the study eye remained stable at 20/20. Baseline color fundus photography, IVFA, and spectral domain optical coherence tomography (SDOCT) images are shown in Fig. 10a–c, respectively. Nonconfocal AOSLO images at 2 months and 7 months show only minor microvascular changes as compared to baseline imaging (Fig. 10d, white arrows), suggesting that the BRVO is well-compensated with no progressive retinal microvasculature changes due to unresolved ischemia. The nonconfocal AOSLO images demonstrate the consistency and repeatability of the technique, and its ability to capture consistent images of the same retinal location over time.

Longitudinal vascular imaging of a 49-year-old female with proliferative diabetic retinopathy is shown in Fig. 11. This patient had a history of diabetes mellitus type 2 controlled with metformin and sitagliptin for 3 years duration. AOSLO imaging at 1° inferior retina of the right eye was done at baseline and 5 months. She received one bevacizumab injection to both eyes 2 months after baseline. During this period, her visual acuity changed from 20/15 to 20/20. Her hemoglobin A1c levels were not available at these times. Baseline color fundus photography, IVFA, and SDOCT images at baseline are shown in Fig. 11a–c, respectively. The fundus photograph and IVFA show evidence of dot-blot hemorrhages and fluorescein leakage with no significant changes in the OCT over the course of 5 months. Two distinct examples of vessel looping are evident at baseline in the confocal AOSLO FA and nonconfocal AOSLO images as revealed by the yellow arrows in Fig. 11d. Full structural regression and loss of capillary patency can be seen at 5 months as indicated by the yellow arrows in Fig. 11e. Neighboring vessels appear to remain intact in the structural images and persistently patent in the perfusion maps over the course of 5 months. Our preliminary results are consistent with a previous study that AOSLO imaging is repeatable and sensitive enough to detect changes such as capillary dropout [43].

#### Limitations of AOSLO for clinical use

Despite the AOSLO’s extraordinary ability to extend our view into the microworld, current instrumentation and protocols have limitations that prevent its widespread clinical use. It is very time-consuming to acquire images, due to its limited field of view, and can be very fatiguing for patients. Furthermore, the optics of the AOSLO exaggerates sensitivity to media opacities, higher refractive errors, fixation stability, and tear film quality, and demands exceptional subject cooperation. Image processing, montaging and analysis are also very labor intensive and time consuming due to lack of automated techniques. Hopefully future advances in speed and tracking will expand its ability to image more challenging eyes, and software advances will make results more rapidly available in a clinically relevant time course.

## Conclusions

AOSLO provides unprecedented views of the retinal vascular network down to the capillary level, revealing microscopic vascular features that are not consistently visible with current clinical ophthalmic imaging instruments. This high resolution imaging technique also shows significant potential for studying physiological and pathological features of the retinal vasculature in the living human eye. Its ability to noninvasively track subclinical vascular changes over time opens new vistas onto the dynamic evolution of certain diseases and provides a more sensitive examination of clinical interventions.

## Abbreviations

AOSLO: adaptive optics scanning light ophthalmoscope
FA: fluorescein angiography
IVFA: intravenous fluorescein angiography
OCTA: optical coherence tomography angiography
SDOCT: spectral domain optical coherence tomography
